# Departure efficiency evaluation of a comprehensive transport hub based on Wi-Fi probe data and a multilayer hybrid model

**DOI:** 10.1371/journal.pone.0264473

**Published:** 2022-03-04

**Authors:** Tangzhi Liu, Jue Shan, Xingliang Liu, Ting Shang

**Affiliations:** College of traffic & transportation, Chongqing Jiaotong University, Chongqing, China; Southwest Jiaotong University, CHINA

## Abstract

Evaluation of the passenger departure efficiency of a comprehensive transport hub is essential for traffic managers. Through the evaluation, security risks in the hub can be found in time to ensure the safe departure of passengers. The attention of existing studies has focused on the analysis of the overall situation of the hub, and the quantitative description of departure status in different connection areas inside the hub is insufficient. In this study, a multilayer hybrid model based on an analytic hierarchy process and entropy weight method was established. The data collected using Wi-Fi probe technology were clustered by a K-means algorithm. The first level of the model was divided according to the connection areas of the passenger hub, and the second level was based on the number of stranded people, wait time and departure time in each connection area. It was found that the SP index has the greatest impact on departure efficiency. In addition, the impact of passenger flow aggregation on each connection area is different, and the management department should treat it accordingly. The applicability of the proposed multilayer hybrid model was verified in the example of the Chongqing north railway station.

## Introduction

With the urbanization and population concentration stemming from social development, the passenger volume of transportation hubs is increasing, as is passengers’ demand for travel comfort and convenience. One of the important functions of a hub is passenger departure. In multitransport hubs, during holiday hours and other peak periods, a significant passenger flow aggregation phenomenon appears. For example, in Chongqing, China, in 2017, the average daily passenger flow through the Chongqing north railway station was 82,000 people, while the peak passenger flow during the Spring Festival on January 26 was 178,000 people, more than twice the average daily passenger flow. Especially when the passenger flow reaches the hub capacity and the departure performance is poor, the large passenger flow phenomenon [1] may lead to serious passenger casualties. Such as the stampede accident due to subway failure at Xidan station in Beijing in 2011. In these cases, hubs face severe challenges in the process of passenger departure. Before taking measures to deal with the passenger flow aggregation phenomena, how to quantify the evacuation of each connecting transportation mode of the hub and comprehensively evaluate the passenger departure efficiency of the overall hub has become an urgent problem to be solved by management departments.

A comprehensive passenger transport hub is a passenger transport site integrated with urban transportation that centrally arranges two or more external transport modes and passenger flow conversion places of urban transportation in the same area [2]. It aims to realize the effective connection of facilities and equipment, transportation organizations, public information and so on. Common connection modes in a passenger hub include rail transit, buses, taxis, coaches and social vehicles. We define the departure efficiency of the hub as the speed at which passengers leave the hub through the connection modes in the hub. Departure efficiency is the most intuitive measure to reflect departure performance and is also the most convenient measure for managers to collect data. It can quantitatively evaluate the passenger flow in hubs of different transport organizations within a given period of time and provide clear guidance for the adjustment of transport capacity under different situations. Therefore, in view of the challenges and safety problems brought by the departure of hub passengers, some studies choose some indicators representing the departure efficiency to establish the index system, including waiting time, processing time, walking time, etc. [3–5]. Analytic hierarchy process [6] (AHP) and fuzzy evaluation [7] are common algorithms used to establish an evaluation index system, which can be verified later in an actual situation. These studies provide basic methods for the evaluation of the flow departure capacity; they help to decompose, compare and integrate the weighted complex passenger flow environment parameters and gradually simplify the mathematical process. Research on evaluation indexes is gradually developing in the direction of systematization. However, at present, the research mainly focuses on the comprehensive evaluation of the overall departure efficiency of the hub [8], and the specific departure conditions in different connection areas still depend on the qualitative analysis of the departure passenger flow. Due to the incorporation of a large number of artificial evaluation processes, the evaluation brings some subjective deviations to the results, which cannot accurately reflect the departure situation. Other studies use computer simulation models to obtain relevant data and simplify the modeling process [9–11], but a certain number of repeated simulations to reduce the deviation is needed [12]. It is also difficult to combine the passenger flow distribution and operation management, which is divorced from reality to a certain extent. That is, there is not enough quantitative research on the departure status of each connection mode, thus making the evacuation measures proposed in the hub less targeted. In contrast, a more quantitative mathematical evaluation model can give more accurate and data-based results, and has a higher reference value for the systematic departure evaluation of the hub.

The main contribution of this study is to solve the above problem of insufficient quantification in hub passenger departure evaluation. A departure efficiency evaluation index system of passenger flow was established through data analysis, and a hybrid quantitative evaluation model was constructed based on an AHP and entropy method to quantitatively evaluate the safety of passenger flow in the departure process.

The remainder of the paper is organized as follows. Section 2 reviews the related research on passenger flow and the evaluation models. From the perspective of passenger flow, in section 3, the connection areas were divided according to different departure modes. Additionally, the method of data collection, Wi-Fi probe technology, is introduced, and the data source of the study is analyzed. In section 4, the clustering results and the weight calculation results of the model are displayed, and the departure efficiency of the Chongqing north railway station is also analyzed. Section 5 concludes the study.

## Research review

In this section, we summarize the relevant studies on the analysis of passenger departure efficiency, select the appropriate detection technology to collect data, use the representative indicators to characterize passenger departure efficiency, and quantitatively evaluate the departure efficiency of hubs.

Jia et al. [13] studied the waiting time of passengers through queuing theory and evaluated hub service efficiency with the goal of minimizing the total cost; Lu et al. [14] constructed a "time-space" network to guide passengers’ outbound behavior. These mainly study the overall departure effect in the hub, but they cannot give targeted evacuation opinions according to different departure modes of passengers. Yao et al. [15] evaluated transfer schemes in the hub from the aspects of passenger satisfaction and comfort; Tao et al. [16] used the same or different expert opinions to reduce the uncertainty of evaluation results; Lai et al. [17] evaluated the hub recovery capacity according to passengers’ travel demand and service quality perception. Although these studies consider passenger transfer in the hub to a certain extent, the analysis is biased toward subjective evaluation and does not give a quantitative rating of the overall departure efficiency of the hub. Therefore, we need a more quantitative model to systematically evaluate the departure performance of passenger flow from the perspective of the overall hub and each connection mode.

In fact, the evaluation accuracy of hub departure efficiency largely depends on the accuracy of the index data. As passenger transport hubs have the characteristics of wide land occupation and large and complex passenger flows, traditional detection technology has difficulty meeting the requirements in terms of detection ability, cost and so on. Automatic fare collection technology (AFC) [18,19], for example, can be utilized only after passengers swipe their card out of the station, resulting in poor real-time performance. Video active detection technology [20,21] is also impractical due to the high cost of cameras and the complex installation of equipment. Infrared sensor technology [22] can accurately calculate the time a passenger passes, but it is difficult to follow passenger movement; thus, detailed space-time dimension data are missing. Additionally, the low recognition rate in dense crowds also limits the use of infrared sensor technology as a detection means in a hub. With the popularity of smartphones [23], Bluetooth technology and Wi-Fi probe technology have increased. However, the limited detection range of Bluetooth technology [24,25] makes it difficult to achieve high-precision positioning. In comparison, Wi-Fi probe technology [26] is superior in its accurate positioning, low cost, lack of additional hardware support and limited effects due to line-of-sight distance and has wide application prospects. It can collect the data of passenger flow in and out of a hub, passenger transfer and the flow density. The collected data can be used for the analysis of passenger evacuation, transfer and other behaviors [27–29].

When selecting the characterization indicators required by the model, the passenger departure efficiency of a passenger station can be reflected by the number of passengers and the time spent from the perspective of passengers [30–33]. First, the number of stranded passengers in a station affects the circulation of the whole hub. Alawad et al. [34] classified the congestion risk of a station into stranded passengers, waiting passengers and train capacity. Sipetas et al. [35] used image processing and video recognition technology to count the passengers stranded at the platform and fused the train operation data to correct the number of passengers in peak hours. Second, the departure time of passenger flow is determined by several factors, including the characteristics of vehicles, passenger boarding and alighting time, vehicle timetables, signals and driver behavior [36]. Models have been established to plan the dwell time of vehicles at a station [37,38]. In addition, for large passenger transport hubs, the passenger wait time in each connection area reflects the collaborative evacuation capacity within the hub. Tesoriere et al. conducted a case study on the movement of users in the departure port of the Fontana Rosa Catania Airport to explore the factors affecting the distribution of airport passenger flow [39]. A passenger itinerary inference model (PIIM) was developed to infer the passenger journey of urban rail transit with high resolution, and the average wait and transfer times of passengers could be estimated as well [40].

To rate the overall departure efficiency of a hub from the situation in different connection areas, several common analytical methods were compared. Factor analysis [41,42], which was first proposed by psychologist C.E. Spearman, was used to calculate the weight through the variance interpretation rate of common factors. However, this kind of calculation method tends to delete redundant evaluation indicators. It can only obtain the weight of each factor but cannot form the weight of the whole system. The AHP [43,44] was proposed by Professor Saaty. It divides various factors in complex problems into interrelated and orderly levels and quantitatively describes the importance of elements at the same level in combination with expert opinions and analysts’ judgment. For instance, Eshtaiwi et al. [45] measured airport performance with 17 key performance indicators. An AHP was applied to calculate the weights selected from three international airports, and suggestions were put forward for the operation management of Libyan airports. Considering the uncertainty of passenger flow in passenger stations, relevant scholars integrate AHP and other evaluation methods. For example, a probability density model [46,47] is more suitable for emergency evacuation than normal passenger flow departures. The combination of an AHP and fuzzy analysis method [48,49] has the disadvantage of strong subjectivity. An entropy weight method [50–52] can measure the uncertainty and use the amount of information carried by the data to calculate the weight, which is biased toward objective calculation and complements the AHP to a certain extent.

For the above analysis, this study constructed a hybrid quantitative evaluation model based on an AHP and entropy method. The hybrid model not only compensates for the disadvantage of the AHP being too subjective but also does not ignore the importance of the index itself, as in the case of using the entropy method alone. The correctness of the method is later tested by application to the Chongqing north railway station.

## Data resources

When a passenger’s Wi-Fi-enabled device enters the effective detection range of the probe and Wi-Fi is turned on, the probe will capture and record the MAC address of the device and generate sequence data with a timestamp (Table 1). At this time, we need to preprocess the data. For example, sometimes a passenger will carry multiple mobile devices, such as standby mobile phones, laptops and tablets, at the same time. To avoid repeated data collection, the collected mobile phone’s MAC address corresponds to the passenger in the station rather than the mobile phone manufacturer. Therefore, we compared the MAC addresses of common mobile phone manufacturers to eliminate terminal devices. In addition, we found that in the original data set collected by a Wi-Fi probe, some MAC addresses appear regularly and frequently over a continuous and long time span but cannot be found in the data set collected by other Wi-Fi probes. In fact, these repeated MAC addresses are those of fixed terminal equipment within the signal coverage or those carried by staff in the station, which need to be identified and eliminated. The identification principles are as follows:

- A MAC address is only detected by one Wi-Fi probe within the hub, and the detection duration exceeds an hour.
- In a week or a month, a MAC address is detected more than 3 times by a Wi-Fi probe at the same detection point, and each detection lasts more than one hour.
- Some MAC addresses match the MAC segment of some fixed terminal equipment manufacturers.

**Table 1:** Data format of the Wi-Fi probe acquisition data.

MAC Address	SN Code	DIS(m)	MAC Attribution	ID	Acquisition time	RSSI
D4:6A:6A:96:B3:9C	MQ1BB83510000020	5.01	Hon Hai Precision Ind. Co., Ltd.	Chongqing Telecom, China	2018/12/30 20:56	-68

In places with dense probes, the MAC address of a terminal device will be detected by different probes at the same time, resulting in the travel position oscillation of the MAC address during detection. Therefore, when the same terminal device is detected by three or more Wi-Fi probes at the same time, the three-point positioning method [47] is used to determine the position of the terminal device.

After data preprocessing, if data loss or abnormal data records occur during the process of data transmission to the cloud, based on a median filter smoothing method, historical data are used to correct the data. Finally, to protect passengers’ privacy, the MAC address data uploaded to the cloud server are desensitized (**Table 2**). The desensitized data will not be associated with the real information of passengers.

**Table 2:** Data format after desensitization.

MAC Address	SN Code	RSSI	Acquisition time
AC:CF:23:##:##:##	MQ2BB85150000169	-88	2019/2/3 0:02

**Fig 1:**
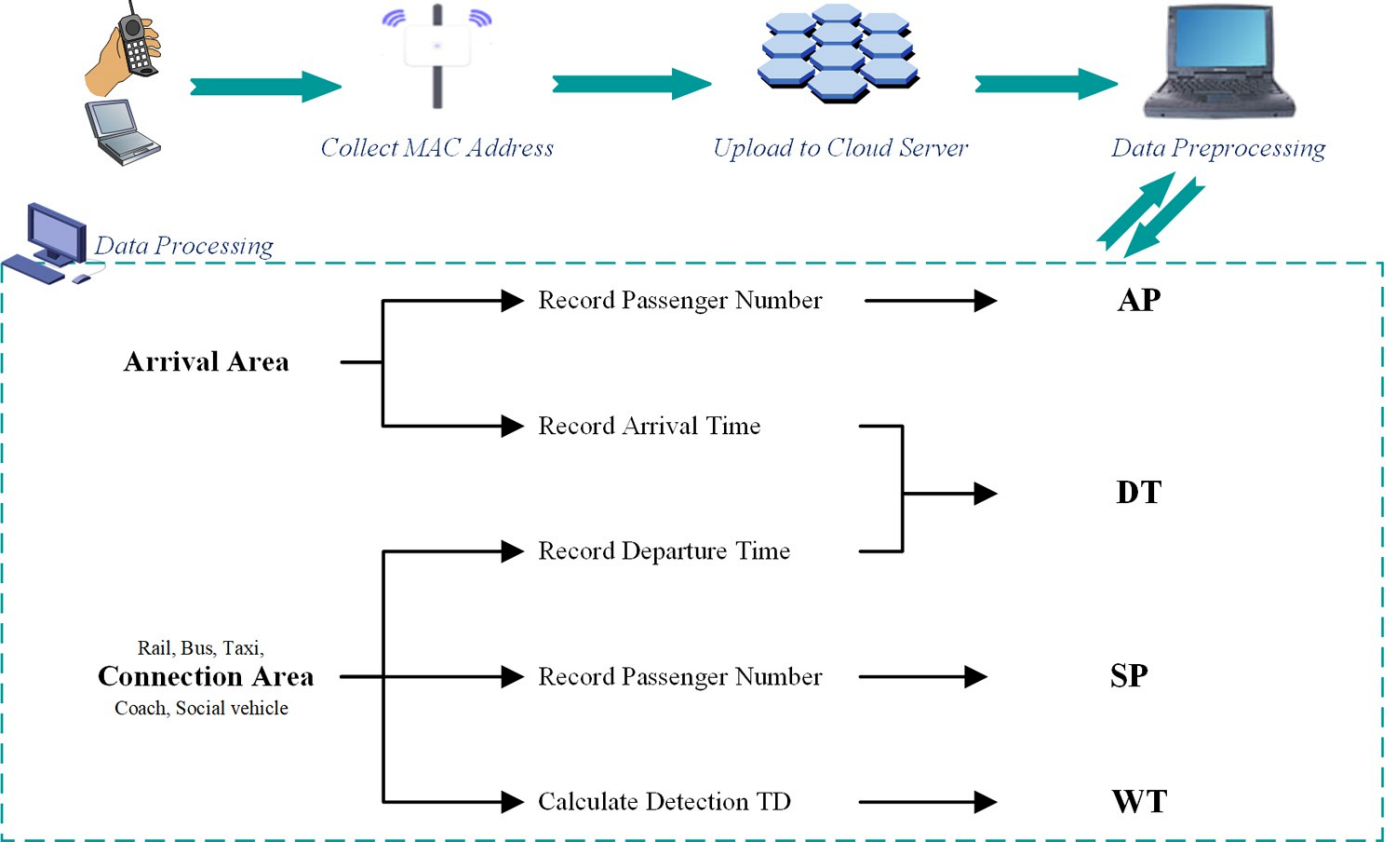
Wi-Fi probe data collection process.

The data collection indicators include the number of arriving passengers (AP), the number of stranded passengers (SP), wait time (WT) and departure time (DT). The data collection process is shown in **Fig 1**.

- The AP reflects the number of new passengers at the station (including pick-up persons). Whether the passenger is an arriving passenger or a receiving passenger is judged according to the retention time of the passenger’s MAC address within the arrival area. An AP is recorded and the sample is expanded. Sample expansion coefficient = total arrivals/detected arrivals.
- The SP is the number of passengers waiting for departure in each connection area. It can be used to reflect the ability to evacuate passengers in different connection areas. The lower or more stable the number is, the better the ability of the connection area to evacuate passengers. According to the stay time of passengers’ MAC address in the connection area, the SP can be counted. The SP in each connection area is recorded and the sample is expanded. Sample expansion coefficient = total stranded/detected stranded.
- The WT is the wait time of passengers in a connection area. This indicates the matching degree of departure demand and capacity level under different connection areas. When the WT is at a relatively stable level, the evacuation capacity of the connection area is at a high match with the station demand in this time period. The WT of passengers within each connection area is recorded.
- Based on the DT, we can analyze the evacuation efficiency of passenger flow leaving the station by selecting different departure modes. If the number is at a relatively stable level for a certain time, the departure path of station passenger flow has good accessibility during the period of time. The DT of passengers within each evacuation function area is recorded.

The processed raw data are output in CSV or TXT format. The output format is shown in **Table 3**.

**Table 3:** Data output format.

**D**ate	**T**ime	**Hub Name**	**Arriving Passengers**				
2019-1-2	09:10	Chongqing north railway station	125				
**Date**	**Time**	**Hub Name**	**Stranded Passengers**				
			**Bus**	**Coach**	**Rail**	**Taxi**	**Social Vehicle**
2019-1-2	09:00	Chongqing north railway station	76	30	106	10	50
**Date**	**Time**	**Hub Name**	**Wait Time/min**				
			**Bus**	**Coach**	**Rail**	**Taxi**	**Social Vehicle**
2019-3-20	09:00	Chongqing north railway station	5.8	27.7	7.0	11.9	15.0
**Date**	**Time**	**Hub Name**	**Wait Time/min**				
			**Bus**	**Coach**	**Rail**	**Taxi**	**Social Vehicle**
2019-3-20	09:00	Chongqing north railway station	18.7	76.8	8.1	12.4	16.0

The data used in this research were collected at the Chongqing north railway station, which is a passenger transport hub dominated by railway transportation and integrates a variety of urban road transportation modes. The Chongqing north railway station is the largest railway station in Southwest China. It applies a passenger information automation system, and the passenger throughput ranks among the top in the country. The station is equipped with a coach connection area, rail transit connection area (lines 4 and 10), bus connection area and taxi connection area. We applied the data to the Chongqing Traffic Planning, Survey, and Design Institute (CSD) because they evenly deployed Wi-Fi probes at this station. The specific points **(Table 4**) and the time period of the required data were submitted to CSD along with the research purpose. Each collection point contains the information of some pick-up people. Since they leave with passengers, these data were retained for the evaluation of departure efficiency. Before the formal trial, we randomly selected data from one working day and one nonworking day for pre-investigation. Comparing the number of arrivals provided by the station and that measured at point 1, it was found that more than 85% of passengers keep their mobile WI-FI on, which ensured the reliability of WI-FI probe technology in this study.

**Table 4:** Layout of data collection points.

Layout Areas	Waiting Collection Point	Departure Collection Point
Arrival Area	point 1	--
Rail Transit Connection Area (Line 4)	point 5	point 6, 13
Rail Transit Connection Area (Line 10)	point 2	point 3, 4
Taxi Connection Area	point 7	point 12
Coach Connection Area	point 8	point 9
Bus Connection Area	point 10	point 11

The formal trial collected 30 days of data collected from September 1, 2019, to September 30, 2019. After data processing, experimental analysis was carried out at an interval of 30 minutes. Considering the daily operation period of the hub, data from 8:00 to 22:00 for each connection area were selected. There were 3 × 4 × 840 groups of data for analysis, with 10,080 groups in total.

**Fig 2:**
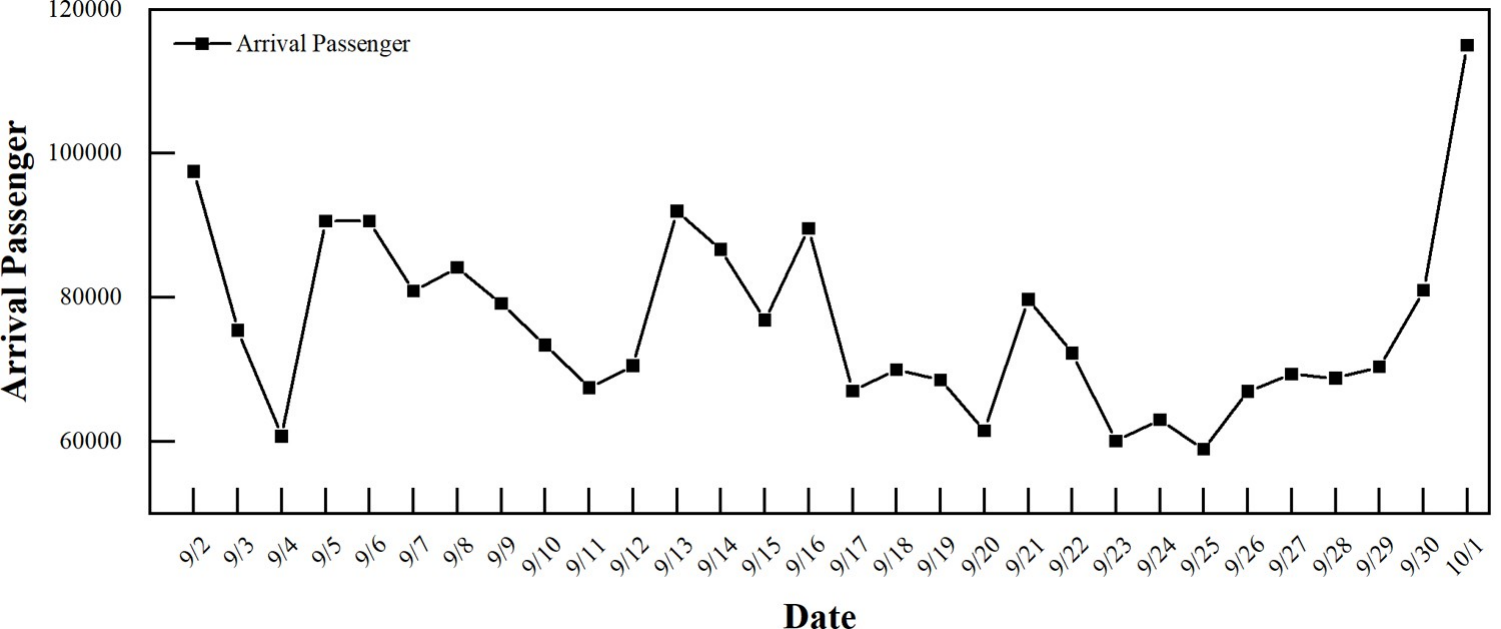
Distribution of the daily arrival passenger flow in the arrival area.

In addition, we found obvious dynamics while analyzing the temporal distribution characteristics of passenger arrivals. Significant daily fluctuations appeared in September (**Fig 2**), reaching a maximum of 115,000 on September 30 (the day before the National Day) and a minimum of 59,000 on September 24 (Tuesday). For the rest of the time period, since Fridays and Sundays are the end of the work week and rest days, respectively, the proportions of arriving passenger flows on Fridays and Sundays are 14.9% and 15.3%, respectively, which are larger than those at other times of the week.

## Model

### Multilayer evaluation index system

As mentioned in section 1, we intend to establish an evaluation index system for passenger departure efficiency. To ensure the integrity and reliability of the system, the selection of evaluation indexes is necessary [53,54]. Analysis shows that the flow of passenger departures in the station is a complex and continuous process, and there is a certain correlation between its operation efficiency, the connection area classification and the feelings of individual passengers. First, as passengers are the main service objects of transportation hubs, their perception and evaluation in the departure process can directly reflect the passenger departure efficiency. Meanwhile, the feelings of passengers in different connection areas are independent of each other. Therefore, the passenger departure evaluation index system can be intuitively divided into two levels. The departure efficiency of the five connection areas is selected as the primary index layer. The SP, WT and DT, as the indicators we collected for passenger comfort [55,56], are selected as the secondary index layer. The passenger departure evaluation index system is shown in **Table 5**.

**Table 5:** Passenger departure efficiency evaluation index system.

Target layer	Primary Index Layer	Secondary Index Layer	Departure Status
Departure Efficiency Evaluation of Passenger Flow in Stations	Rail Transit Connection Area Departure Efficiency (A1)	SP (B1)	Level 1—Level 5
		WT (B2)	Level 1—Level 5
		DT (B3)	Level 1—Level 5
	Conventional Bus Connection Area Departure Efficiency (A2)	SP (B4)	Level 1—Level 5
		WT (B5)	Level 1—Level 5
		DT (B6)	Level 1—Level 5
	Taxi Connection Area Departure Efficiency (A3)	SP (B7)	Level 1—Level 5
		WT (B8)	Level 1—Level 5
		DT (B9)	Level 1—Level 5
	Coach Connection Area Departure Efficiency (A4)	SP (B10)	Level 1—Level 5
		WT (B11)	Level 1—Level 5
		DT (B12)	Level 1—Level 5
	Social vehicle Connection Area Departure Efficiency (A5)	SP (B13)	Level 1—Level 5
		WT (B14)	Level 1—Level 5
		DT (B15)	Level 1—Level 5

The division and basic characteristics of the evaluation index corresponding to the passenger departure state are described in **Table 6**.

**Table 6:** Description of departure state levels corresponding to each evaluation index.

Evaluation Index	Departure Status	Basic Feature
SP	Level 1	Almost none, passengers come and go immediately
	Level 2	Very few, passengers basically go as they arrive
	Level 3	Few, difficult for passengers to flow
	Level 4	Much, few passengers gather
	Level 5	Many, mass passenger gathering
WT	Level 1	Almost none, passengers come and go immediately
	Level 2	Very short, passengers basically go as they arrive
	Level 3	Relatively short, difficult for passengers to flow
	Level 4	Long, few passengers gather
	Level 5	Very long, mass passenger gathering
DT	Level 1	Almost none, passengers can leave the station in a short time
	Level 2	Relatively short, passengers can leave the station in a relatively short time
	Level 3	Relatively long, sporadic passenger gathering at the station
	Level 4	Long, a general passenger gathering at the station
	Level 5	Very long, mass passenger gathering at the station

Additionally, in **Fig 2** of section 2, we can clearly see that the characteristics of passenger flow are dynamic over time. The requirements for the efficiency of passenger departure stations are different in different periods. If a traditional clustering method based on division is used to process passenger flow data, the error is large, and the amount of calculation is considerable. Therefore, a K-means algorithm is used to cluster the secondary index layer. According to the evaluation indexes in **Table 3**, the cluster center $C^{\left(t\right)}=(c_{1}{}^{\left(t\right)},c_{2}{}^{\left(t\right)},c_{3}{}^{\left(t\right)},c_{4}{}^{\left(t\right)},c_{5}{}^{\left(t\right)})$ is obtained, which determines the interval range of passenger flow under different departure states. This realizes the specific quantification of the passenger flow departure states. We define $c_{1}{}^{\left(t\right)},c_{2}{}^{\left(t\right)},c_{3}{}^{\left(t\right)},c_{4}{}^{\left(t\right)},c_{5}{}^{\left(t\right)}$ as cluster centers at all levels and *c*_max_ as the maximum value in historical data. The calculation of the departure state interval division is shown in **Eq (1)**.


$$\begin{array}{l} \mathrm{Level}\;1\;\mathrm{departure}\;\mathrm{status}\;\mathrm{interval}:\;(0,\frac{c_{1}{}^{\left(t\right)}+c_{2}{}^{\left(t\right)}}{2}]\\ \mathrm{Level}\;2\;\mathrm{departure}\;\mathrm{status}\;\mathrm{interval}:\;(\frac{c_{1}{}^{\left(t\right)}+c_{2}{}^{\left(t\right)}}{2},\frac{c_{2}{}^{\left(t\right)}+c_{3}{}^{\left(t\right)}}{2}]\\ \mathrm{Level}\;3\;\mathrm{departure}\;\mathrm{status}\;\mathrm{interval}:\;(\frac{c_{2}{}^{\left(t\right)}+c_{3}{}^{\left(t\right)}}{2},\frac{c_{3}{}^{\left(t\right)}+c_{4}{}^{\left(t\right)}}{2}]\\ \mathrm{Level}\;4\;\mathrm{departure}\;\mathrm{status}\;\mathrm{interval}:\;(\frac{c_{3}{}^{\left(t\right)}+c_{4}{}^{\left(t\right)}}{2},\frac{c_{4}{}^{\left(t\right)}+c_{5}{}^{\left(t\right)}}{2}]\\ \mathrm{Level}\;5\;\mathrm{departure}\;\mathrm{status}\;\mathrm{interval}:\;(\frac{c_{4}{}^{\left(t\right)}+c_{5}{}^{\left(t\right)}}{2},c_{\max}] \end{array}$$ (1)

### Multilayer hybrid evaluation model

From section 4.1, we can determine the interval range $\left[x_{ij}^{L},x_{ij}^{U}\right]$ of the departure efficiency evaluation index data under different departure states. The range of passenger flow departure states corresponding to the evaluation index is transformed into intuitionistic fuzzy numbers. Intuitionistic fuzzy entropy theory is used to calculate the weight of each index in the secondary layer. Generally, the higher the departure status level of a certain connection mode at the current time, the lower the weight value of the secondary index, indicating that the departure efficiency of this mode under this index is worse. The weight value of the primary indicators is determined by expert scoring and calculated by an AHP to reduce the influence of human factors. The higher the weight value is, the more important the mode. After comprehensive calculation, the evaluation model of the departure efficiency at the station is constructed.

We define *x*_*ij*_ as the *i*^*th*^ value under the *j*^*th*^ evaluation index in the passenger flow data sample, *m* as the number of samples in the evaluation index data set, and *n* as the number of evaluation indicators in a single data sample. The evaluation index data set of passenger departures is defined in **Eq (2)**.


$$X={\left\{x_{ij}\right\}}_{m\times n}={\left\{\begin{array}{ccccc} x_{11} & \cdots & x_{1j} & \cdots & x_{1n}\\ \vdots & \ddots & \vdots & \ddots & \vdots \\ x_{i1} & \cdots & x_{ij} & \cdots & x_{in}\\ \vdots & \ddots & \vdots & \ddots & \vdots \\ x_{m1} & \cdots & x_{mj} & \cdots & x_{mn} \end{array}\right\}}^{T}$$ (2)

Fuzzy entropy is based on information entropy and was proposed through fuzzy theory to measure the fuzziness of fuzzy sets. The weight value of each secondary index can be calculated based on information entropy. The lower the information entropy is, the smaller the dispersion of the index data, the more information can be provided, and the smaller the weight value of the index is, the greater the impact of the index on the comprehensive evaluation.

Defining y_*ij*_ as the standardized indicator data, the information entropy S_*j*_ of each evaluation index is calculated using **Eq (3)**. The weight *w*_*j*_ of each index in the secondary index layer is calculated using **Eq (4)**.


$$S_{j}=-\frac{1}{\ln m}\sum_{i=1}^{m}y_{ij}\ln y_{ij}$$ (3)

$$w_{j}=\frac{1-S_{j}}{n-\sum_{j=1}^{n}S_{j}}$$ (4)

The evaluation of the departure efficiency based on the connection area was carried out according to the following steps.

1. Establish a factor set for evaluating the efficiency of passenger departure
  The evaluation factor set contains all the evaluation indexes in the evaluation index system, which has the characteristics of stratification. The primary evaluation index *X* = {*x*_1_,*x*_2_,⋯,*x*_*n*_} represents each connection area, and the secondary evaluation index contains three factor indexes: SP, WT and DT.
2. Determine the departure status grade of the secondary layer index
  According to the historical passenger flow data collected by the passenger flow data collection system, the historical passenger flow data are clustered by the K-means algorithm (**Eq (1)**), and the interval range of the passenger flow departure status grade of the secondary evaluation index is divided.
3. Determine the weight vector of the comprehensive evaluation of the passenger departure efficiency
  For the primary evaluation index based on the connection mode, the AHP is adopted to determine the weight of each mode in the departure efficiency evaluation, considering their impact on the passenger flow departure efficiency. The weight vector of the first level index layer *W*_*i*_ = [*w*_1_,*w*_2_,⋯,*w*_*i*_,⋯,*w*_*n*_]^*T*^ is calculated. For the secondary evaluation index, the entropy weight method is used to calculate its information entropy, and then the weight vector of the secondary index *W*_*ij*_ = [*w*_*i*1_,*w*_*i*2_,⋯,*w*_*ij*_,⋯,*w*_*in*_]^*T*^ is determined.
4. Calculate the evaluation index value of passenger flow data
  - Standardized processing of index data

Passenger flow is divided into revenue type and cost type according to the departure state interval in **Eq (1)**, and the index data are standardized. Define *k*_*ij*_ as the passenger departure status level of the *i*^*th*^ passenger flow data under the *j*^*th*^ evaluation index in the data. When the evaluation index is a revenue type index, it is defined by **Eq (5)**.


$$z_{ij}=\frac{x_{ij}-x_{{}_{ij}}^{L}}{x_{{}_{ij}}^{U}-x_{{}_{ij}}^{L}}+(k_{ij}-1)$$ (5)

When the evaluation index is a cost index, it is defined by **Eq (6)**.


$$z_{ij}=\frac{x_{{}_{ij}}^{U}-x_{ij}}{x_{{}_{ij}}^{U}-x_{{}_{ij}}^{L}}+(k_{ij}-1)$$ (6)

b. Calculate the weighted average value of the secondary index layer

Define *Z*_*i*_ as the weighted average value of the evaluation indexes of the secondary index layer by **Eq (7)**, *z*_*ij*_ is the standardized evaluation index data.


$$Z_{i}=\sum_{j=1}^{n}w_{ij}z_{ij}$$ (7)

5. Calculate the evaluation results of the evacuation efficiency level

The decision comment set contains various possible evaluation results of the evaluation index object, recorded as *V* = {*v*_1_,*v*_2_,⋯,*v*_*i*_,⋯,*v*_*n*_}. The evaluation results of departure efficiency *v*_*i*_ can be defined using **Eq (8)**. It is obtained from the determined weight vector of the primary index layer, the weighted average of the secondary index layer evaluation indexes and the grade evaluation set of the passenger flow departure efficiency.


$$v_{i}=\sum_{i=1}^{n}w_{i}Z_{i}$$ (8)

The evaluation level of the departure efficiency is divided into five levels through standardized methods, as shown in **Table 7**.

**Table 7:** Passenger flow departure efficiency evaluation classification criteria.

	Level 1	Level 2	Level 3	Level 4	Level 5
**Departure Efficiency**	Excellent	Good	Commonly	Poor	Bad
**Index Range**	(0,1]	(1,2]	(2,3]	(3,4]	(4,5]

**Fig 3:**
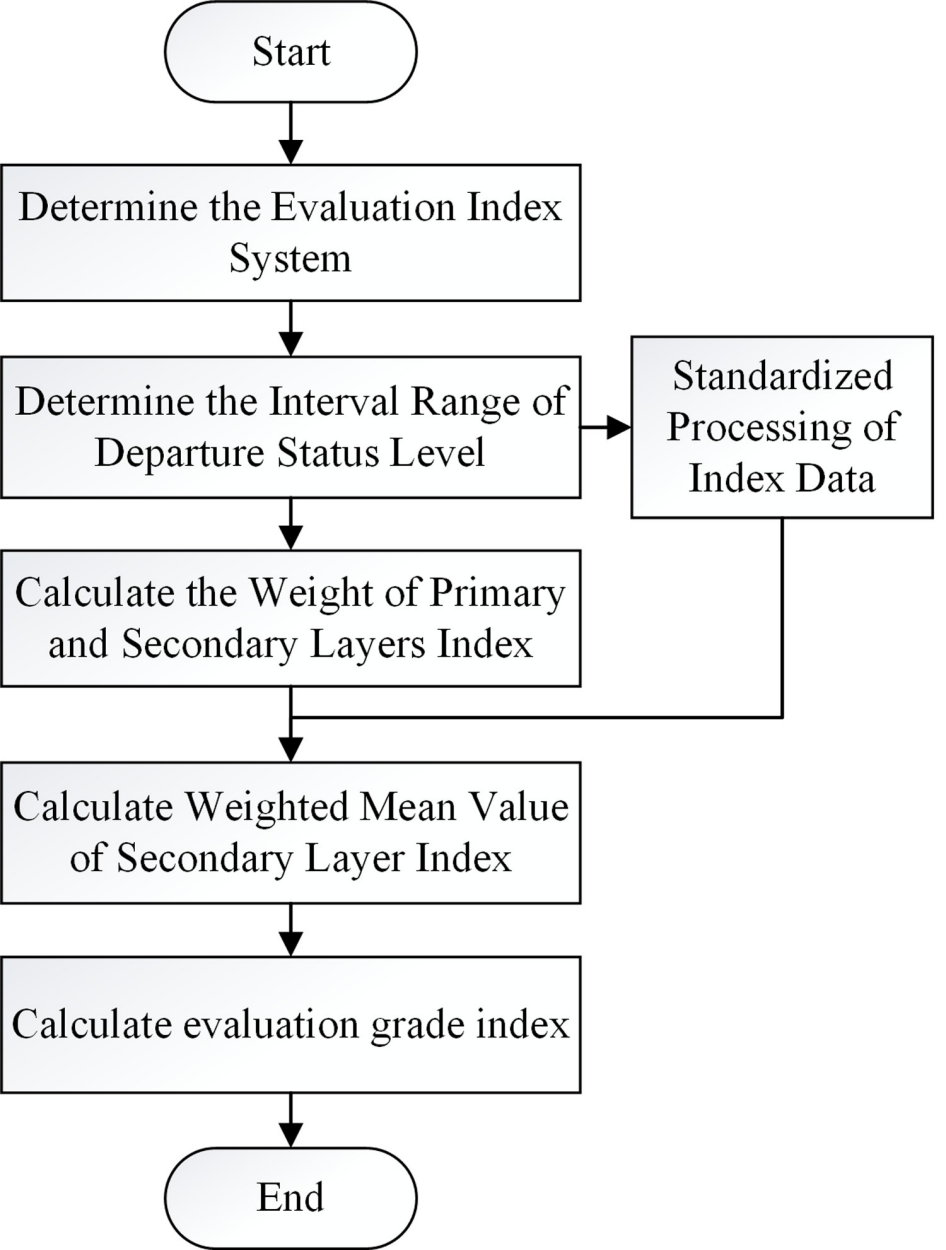
Standardized flow chart for evaluating the efficiency of passenger departure.

The standardized process of evaluating the efficiency of hub passenger departure stations is shown in **Fig 3**.

## Results

### Clustering results

**Fig 4:**
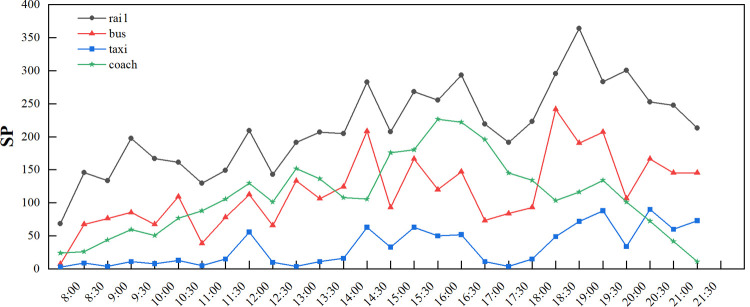
Time-varying chart of the index data of each connection area on September 30 (SP).

**Fig 5:**
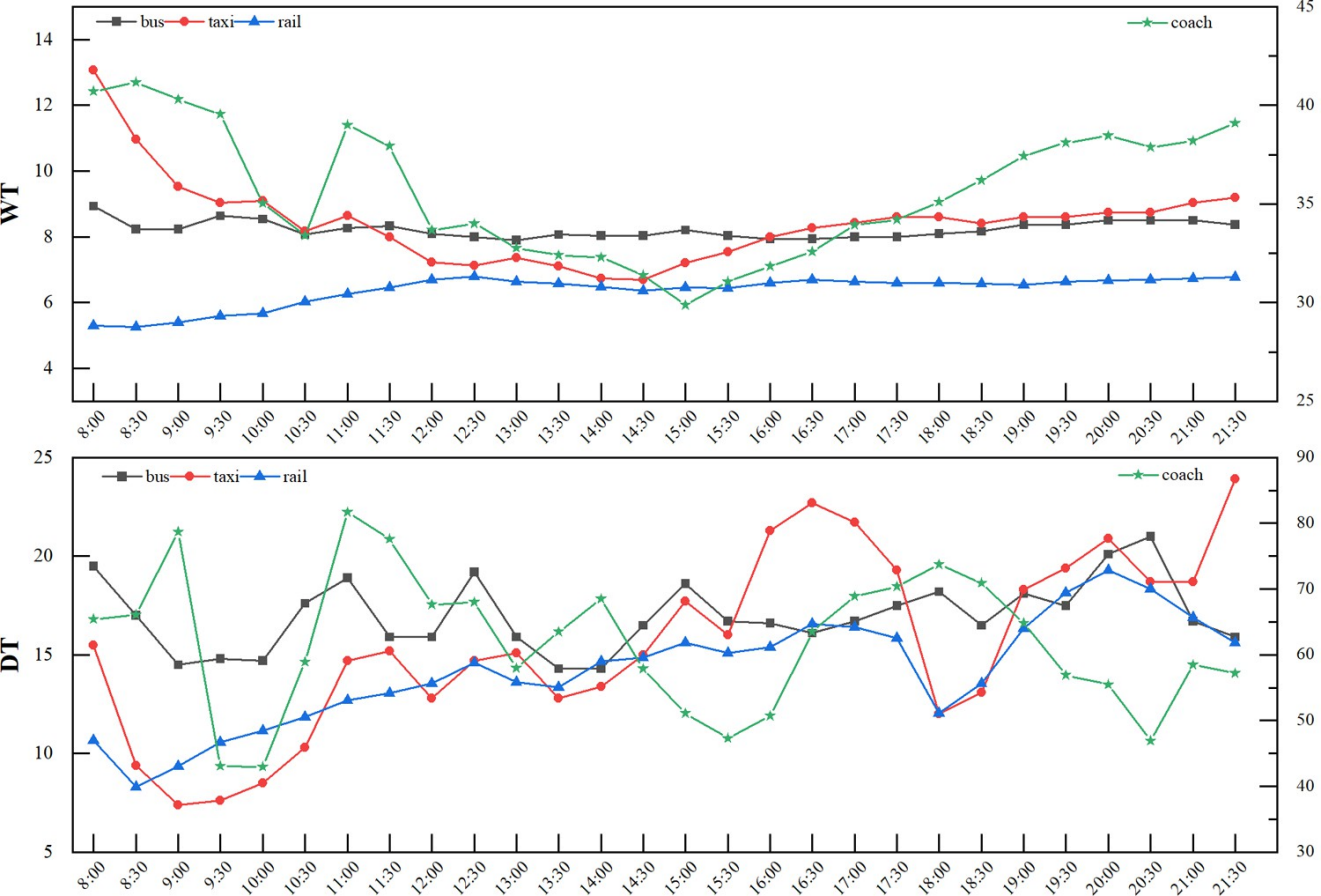
Time-varying chart of the index data of each connection area on September 30 (WT, DT).

Before clustering, we take data on September 30, 2019, as an example to preanalyze the single-day data of each connection area (**Figs 4 and 5**). The detailed data are described in the supporting information (**S1 Table**). Compared with other modes, the number of stranded people in the coach connection area is at a medium level, while the wait time and departure time are much longer than those in other areas. The rail transit connection area has the largest passenger flow, but data show that the time spent by passengers transferring in this area is not long. The three values of the taxi area are all relatively small.

**Fig 6:**
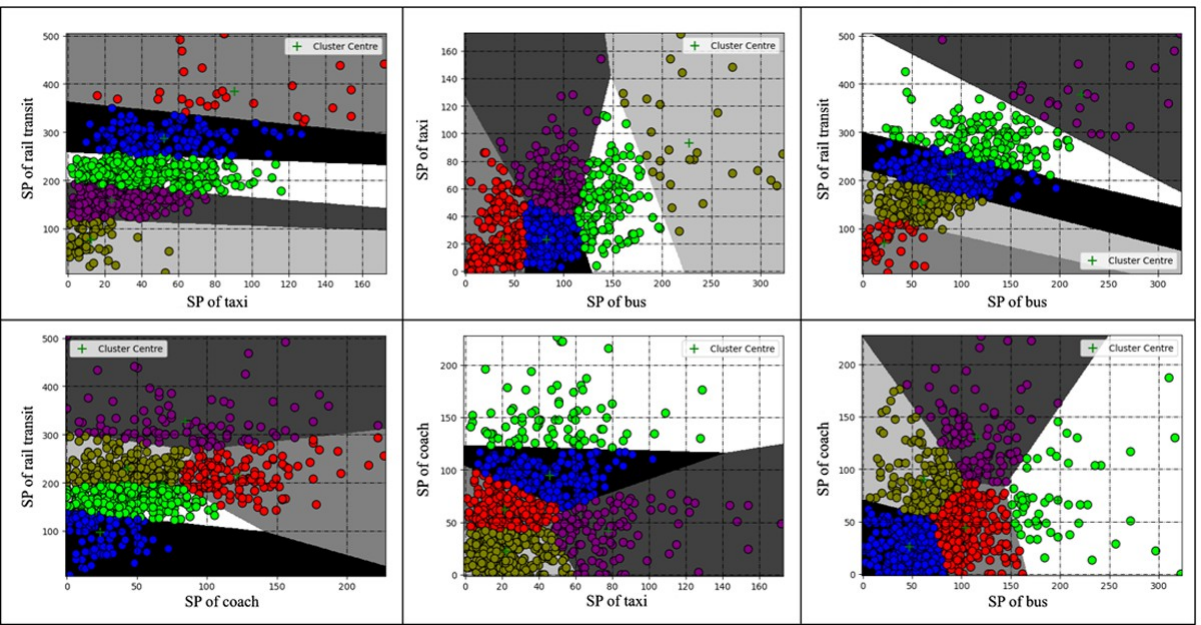
SP cluster analysis results.

**Fig 7:**
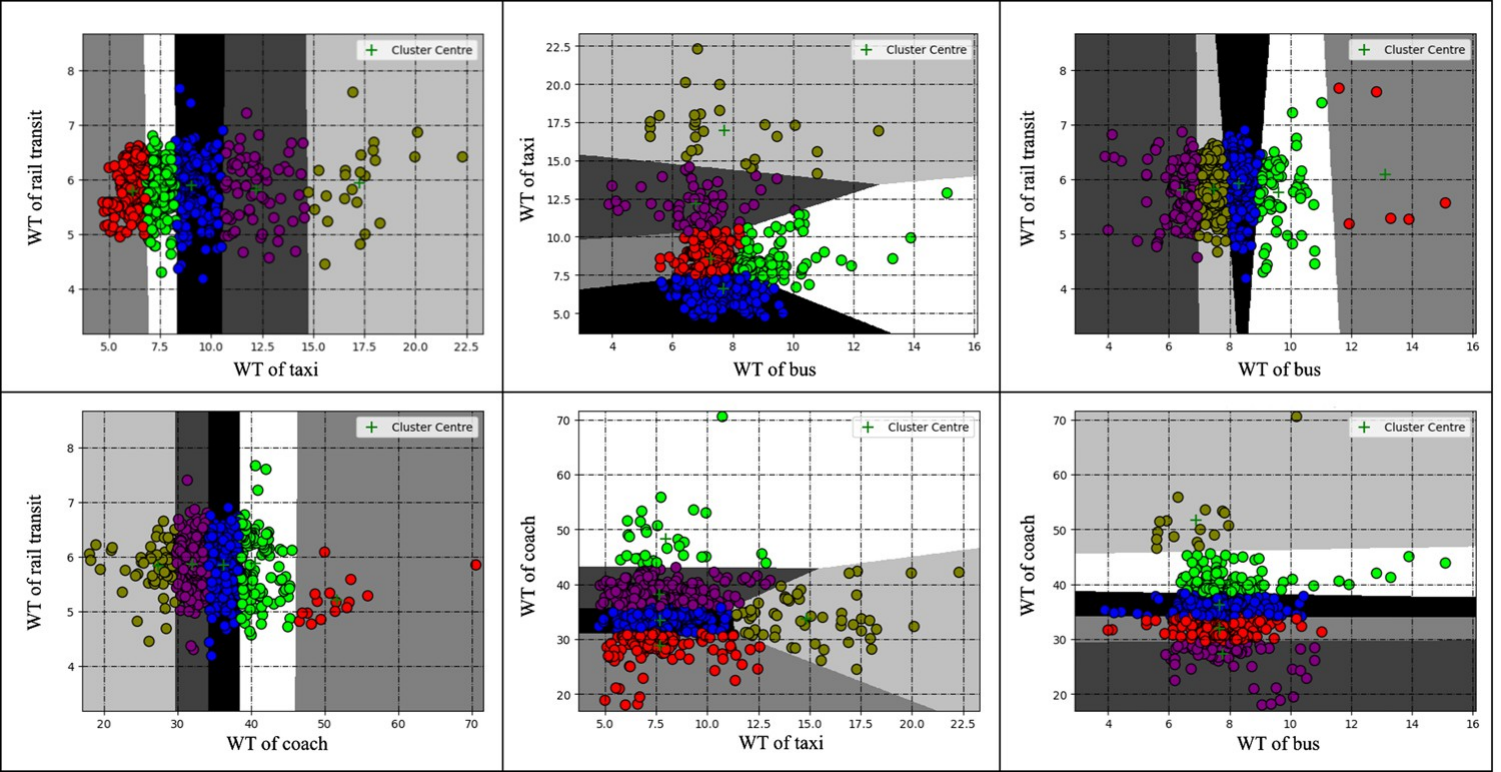
WT cluster analysis results.

**Fig 8:**
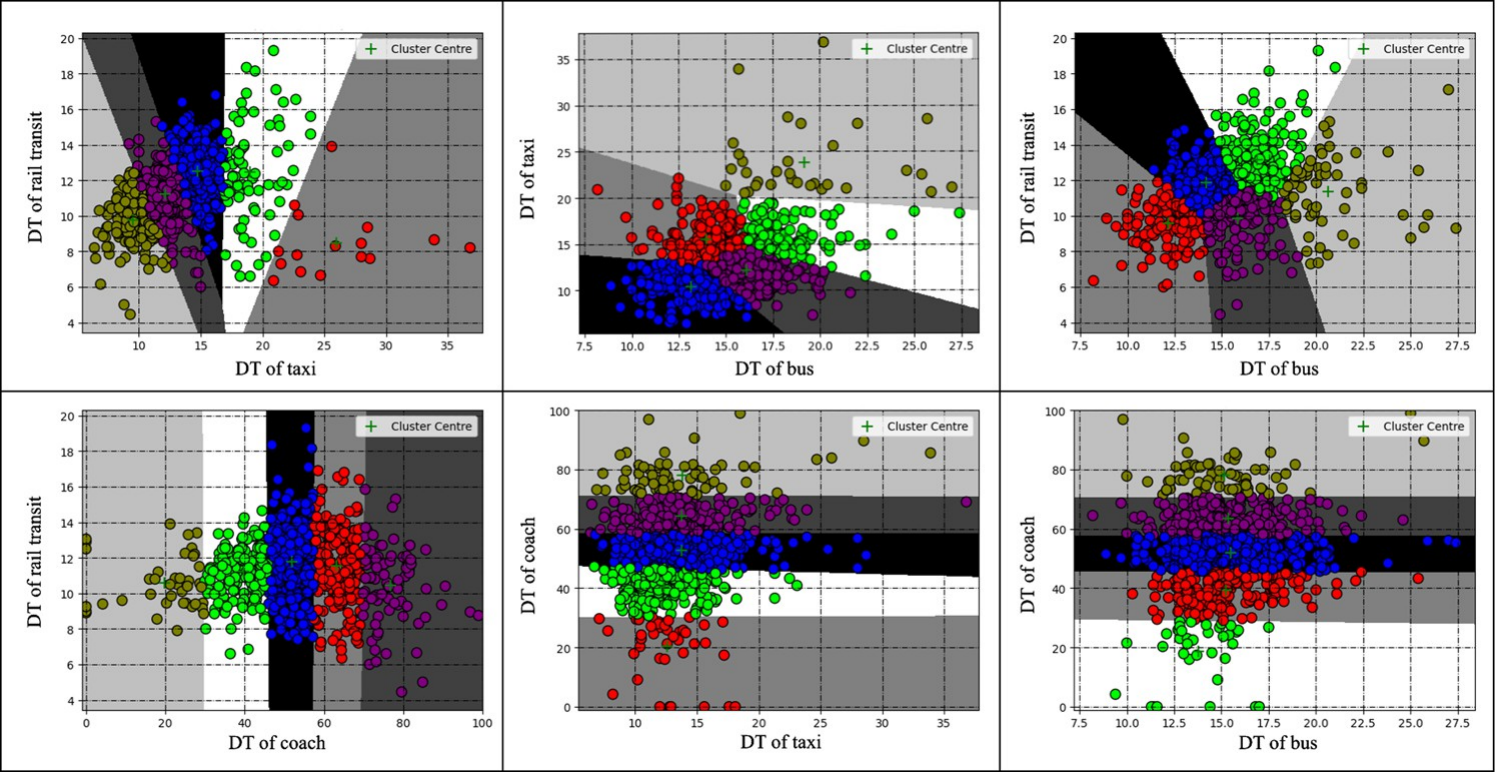
DT cluster analysis results.

Using Python as the platform, the k-means clustering of passenger flow data was realized through programming. Since the social vehicle connection area on the second floor had not been fully put into use during the research, its temporary parking lot spaces were not yet within the connection area. Therefore, we only consider the four connection modes in this study. The historical data set in September is clustered as shown in **Figs 6–8**, and the fuzzy interval ranges of departure state $\left[x_{ij}^{L},x_{ij}^{U}\right]$ corresponding to the SP, WT and DT are determined as shown in **Table 8**.

**Table 8:** Range of passenger departure states.

Index Layer		Departure State Level				
Primary	Secondary	Level 1	Level 2	Level 3	Level 4	Level 5
A1 (Rail)	B1	(0,113]	(113,185]	(185,250]	(250,334]	(334,600]
	B2	(0,5.75]	(5.75,5.81]	(5.81,5.88]	(5.88,6.1]	(6.1,8]
	B3	(0,9.5]	(9.5,11]	(11,12]	(12,13]	(13,15]
A2 (Bus)	B4	(0,41]	(41,76]	(76,109]	(109,174]	(174,350]
	B5	(0,7]	(7,8]	(8,9]	(9,11]	(11,16]
	B6	(0,13]	(13,15]	(15,17]	(17,19]	(19,25]
A3 (Tax)	B7	(0,18]	(18,33]	(33,47]	(47,72]	(72,140]
	B8	(0,6.5]	(6.5,8.3]	(8.3,10.5]	(10.5,14.5]	(14.5,20]
	B9	(0,11.5]	(11.5,14]	(14,17.5]	(17.5,22.5]	(22.5,30]
A4 (Coach)	B10	(0,35]	(35,58]	(58,81]	(81,110]	(110,240]
	B11	(0,30]	(30,35]	(35,39]	(39,46]	(46,50]
	B12	(0,30]	(30,46]	(46,58]	(58,71]	(71,80]

Notes: We use people/30 minutes as the unit of SP, and minutes as the unit of WT and DT.

In combination with **Figs 6–8** and **Table 8**, the comparison of cluster centers in each connection area is as follows. SP: rail>bus>coach>taxi; WT: rail<bus<taxi<coach; DT: rail<bus<taxi<coach. From the overall clustering results, the passenger flow remains relatively unblocked in the Chongqing north railway station since data within level 5 are less scattered. From the perspective of each connection area, the clustering of the three indicators of the taxi connection area is relatively consistent and basically concentrated in level 1, level 2 and level 3; The number of stranded passengers in the coach area is concentrated in level 1, level 2 and level 3, while the departure time in the area is concentrated in level 2, level 3 and level 4. The grades of the three indicators in the track area are relatively high. The clustering results are consistent with the actual passenger flow.

### Weight calculation results

As the dynamism of data refers to in section 2, the study evaluates the departure efficiency of the hub on a daily basis. Taking the real-time passenger flow data collected on September 30 as an example, 28 sample data representing the departure efficiency of each connection area from 8:00 to 22:00 were selected to form the original evaluation index data set *X*(*t*). Through an expert questionnaire survey, according to the 1~9 scale method of the AHP, the importance degree of the departure efficiency targeted at the selection of connection mode is scored, and the weight of each mode is calculated. The consistency of the judgment matrix was tested, and the calculation results of the AHP are shown in **Table 9**.

**Table 9:** Judgment matrix and weight calculation results of the primary index.

Indicator	Judgment Matrix				*W*_*i*_	Consistency Test
Rail	1	5	3	9	0.562	CR = 0.074<0.1, Pass
Bus	1/5	1	1/3	5	0.129	
Taxi	1/3	3	1	6	0.266	
Coach	1/9	1/5	1/6	1	0.043	

After the transfer mode weight of the primary index *W*_*i*_ was calculated, the weights of the secondary indicators *W*_*ij*_ were also calculated. The taxi connection area evaluation index data set and the corresponding interval were input. The entropy weight ranges of the SP, WT and DT were defined using the entropy weight method. According to the optimization theory of linear programming, the weight of the secondary indicators in the four connection areas is shown in **Table 10**.

**Table 10:** Weight calculation results of the secondary indicators.

*w*_*ij*_	SP	WT	DT
Rail	0.496	0.140	0.364
Bus	0.935	0.004	0.061
Taxi	0.547	0.045	0.408
Coach	0.695	0.243	0.062

According to **Table 9**, the importance degree of the four modes decreases as follows: rail, taxi, bus and coach. Among them, the weight of rail transit is the highest, with a value of more than 0.5, while the weight of coach is the lowest. This result is consistent with the functional division of the Chongqing north railway station and the actual operation of the station reflected in **Fig 4**.

However, in the comparison of the four evaluation indexes in **Table 10**, the proportion of the SP index is the largest, followed by the DT and finally the WT. Combined with the interpretation of each index in the third section, the main index affecting the departure efficiency of each connection area is the SP. In addition, the weight value of the bus index SP reaches 0.935, which shows that the evacuation capacity of the bus station is almost completely determined by the number of stranded people at the platform. This phenomenon is mainly caused by the fixed departure interval and seating rate of buses and the characteristics of arriving and leaving immediately. In this regard, although rail transit is similar, due to the large single traffic volume, the impact of stranded people on rail transit is much smaller. Since the departure interval of the coach area is longer than that of the other modes, when passengers choose coaches for connection, the matching degree between the departure time and the arrival time of the main passenger flow is more important; thus, the WT indicator of the coach area also accounts for a larger proportion. In contrast, there is no fixed departure interval for taxis, the accessibility requirements are much higher than other methods, and the corresponding DT weight becomes larger.

### Results of the departure efficiency evaluation

**Fig 9:**
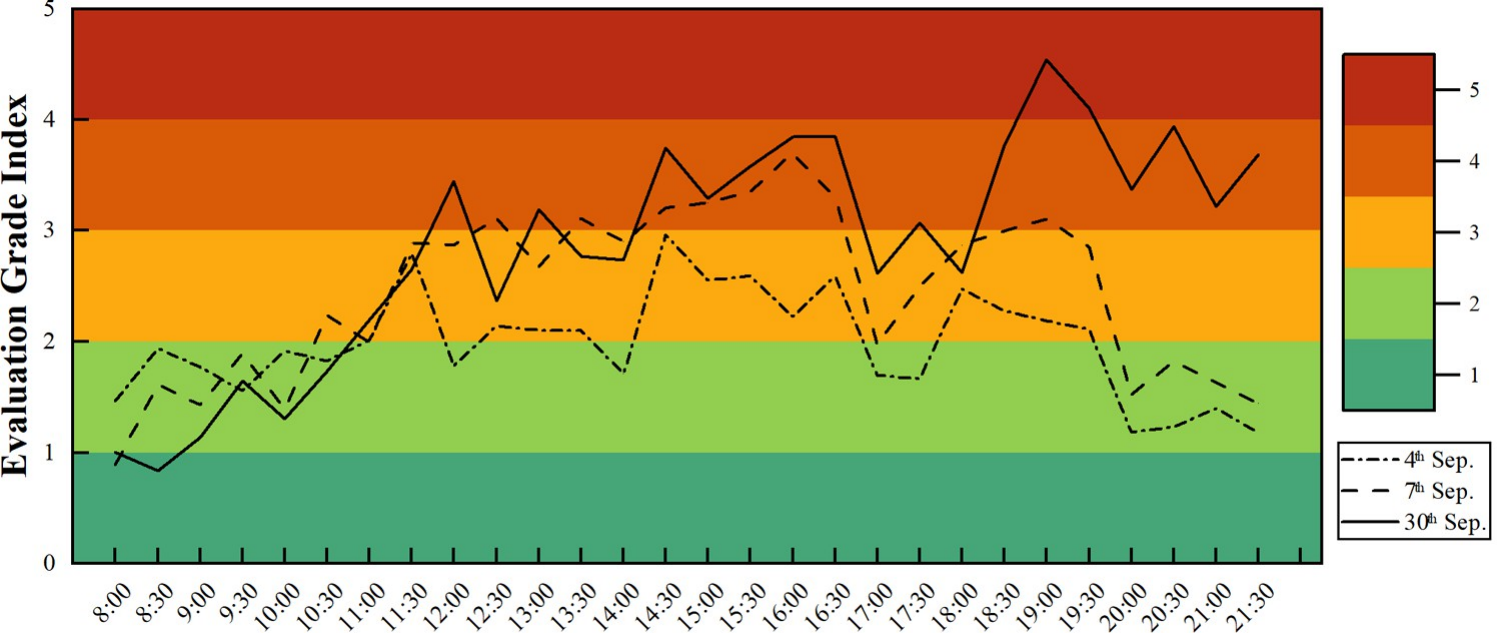
Evaluation grade index diagram of the passenger flow departure efficiency in the north square.

After the departure evaluation results *v*_*i*_ of the station on September 30 were calculated, the data of September 4 (working day) and September 7 (nonworking day) were selected for comparison. The index diagram is shown in **Fig 9**.

It can be seen from the above figure that the departure efficiency at the Chongqing north railway station has an obvious time distribution, and the overall departure level of passenger flow is concentrated at level 2 and level 3. In other words, the passenger evacuation capacity of the Chongqing north railway station is above the medium level, which is consistent with our investigation. On September 4 (working day), the evaluation grade index basically fluctuated up and down at 2.0 and the range did not exceed two grades. On September 30 (the day before the National Day), the peak of the index once exceeded 4.0, reaching the level of poor departure efficiency, but then decreased. That is, although there is a large instantaneous increase in passenger flow to the station before holidays, the overall departure capacity of the station is good. In contrast, the change trend of the evaluation grade index on nonworking days is roughly the same as that before holidays in the daytime, and the fluctuation range did not exceed one grade. The difference was reflected after 17:30. We find that the variance of the evaluation grade index during holidays is 1.03, the variance of the evaluation grade index during nonworking days is 0.62, and the variance of the evaluation grade index during working days is 0.23, indicating that the evaluation grade index fluctuates more during holidays, which is closely related to the sudden increase of passenger flow to the station in the station when holidays are approaching.

**Fig 10:**
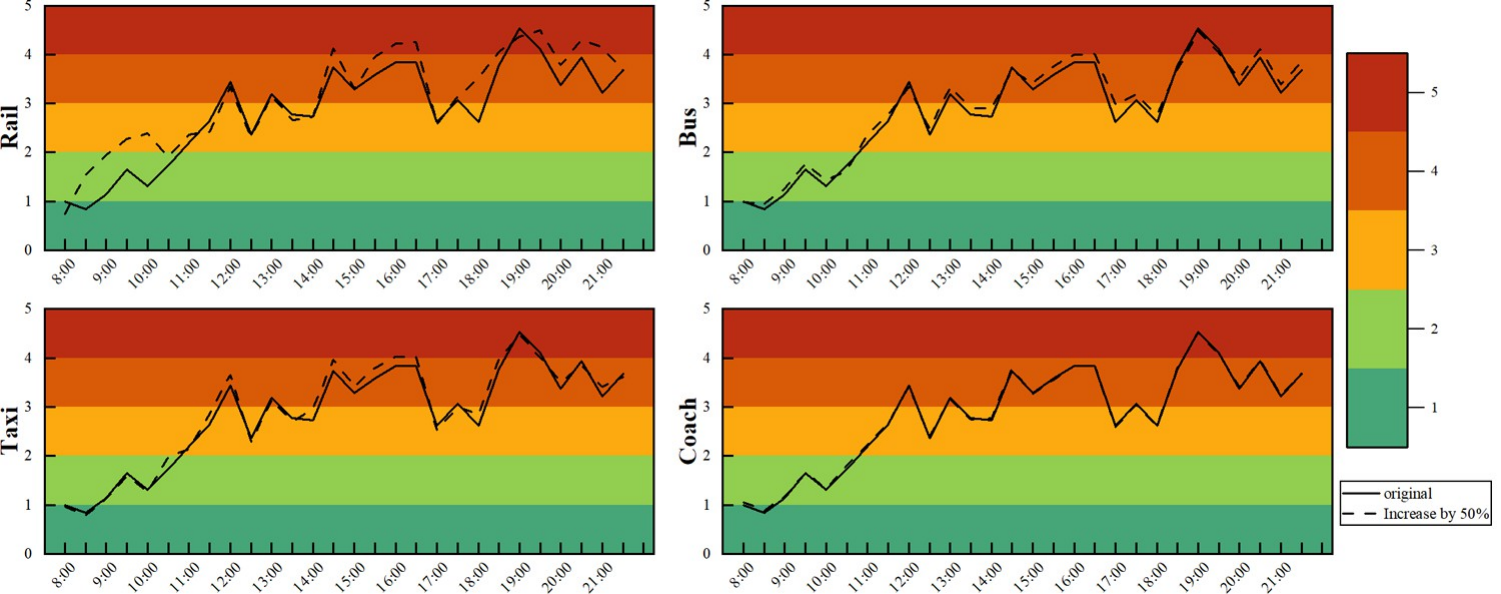
Comparison chart of the passenger flow efficiency evaluation in each connection area.

At present, the large passenger flow phenomenon is a hot issue in the field of public transport. It usually occurs in the comprehensive transportation hub in the case of holidays or sudden accidents. There is an upper limit on the capacity of a station to accommodate passengers. Once the passenger flow reaches saturation, passengers will experience psychological discomfort, and the whole passenger flow group will walk slowly and crowd [57]. We use the passenger flow data on September 30, 2019 (the day before the National Day), to evaluate the passenger flow departure efficiency under the condition of large passenger flow. The number of stranded people in the rail, bus, taxi and coach connection areas in the evaluation index was increased by 50% to simulate the arrival of a sudden large passenger flow. The evaluation index data were brought into the evaluation model, and the impact on the overall departure efficiency under the condition of large passenger flow in different connection areas was obtained through calculation. The calculation results of the passenger departure efficiency evaluation grade index under the condition of large passenger flow are shown in **Fig 10**.

When large passenger flow gatherings occur in different connection areas of the North Square, the impact on the overall departure efficiency is different, in the order of rail > bus > taxi > coach. When the number of stranded passengers in the rail transit connection area increases by 50%, it has the greatest impact on the overall passenger flow departure efficiency. In the case of a large passenger flow, the evaluation grade index of the departure efficiency increases by 14% on average. When the stranded passenger flow in the conventional bus connection area increases by 50%, the evaluation grade index of the departure efficiency increases by 4% on average. When the stranded passenger flow in the taxi connection area increases by 50%, the evaluation grade index of the departure efficiency increases by 2% on average. When the stranded passenger flow in the coach connection area increases by 50%, it has the least impact on the overall departure efficiency of passenger flow, and the evaluation grade index of the departure efficiency increases by only 1% on average.

## Discussion and conclusions

In this study, a hybrid model based on an AHP and entropy weight method is proposed, to evaluate the evacuation capacity of the overall hubs and of each connection mode inside the hub.

For the hubs with dense passenger flow, the data can be collected by Wi-Fi probes. After preprocessing, the evaluation indexes affecting the passenger flow departure efficiency can be extracted, including the number of arriving passengers (AP), the number of stranded passengers (SP), the wait time (WT) and the departure time (DT). When evaluating the departure efficiency of a hub and designing solutions, traffic managers should comprehensively consider both the capacity supply of the hub and the demand of passengers. For example, Alawad’s model mentioned in section 2, that selects indicators related to the number of passengers such as stranded passengers, is more suitable for objectively assessing the risk of stations.

The following conclusions can be obtained from the case study of the Chongqing north railway station: (1) A K-means algorithm can be used to cluster the stranded passengers, wait time and departure time of each connection area in the hub. A total of 10,080 groups of data, including working days and holidays in a month, were selected to cluster the passenger departure status into five levels. The clustering results showed that the passenger flow of the Chongqing north railway station is relatively smooth, and the passenger flow evacuation capacity of each connection area is different. (2) The SP of each connection area has the greatest impact on its departure efficiency when calculating the weight of each index. When the departure efficiency is divided into five levels, the model results showed that the departure efficiency of the Chongqing north railway station is at levels 2 to 4 as a whole on the research days. Among them, the evaluation level on working days fluctuated up and down at level 2, and the weekend days rose slightly. During holidays, the passenger flow increased significantly, and the departure efficiency worsened. (3) We increased the SP index of each connection area by 50% and further explored the passenger flow evacuation efficiency under the influence of a large passenger flow. Large passenger flow aggregation had the least impact on the bus connection area and the greatest impact on the rail connection area. (4) From the result data, the phenomenon of large passenger flow has a greater effect on the departure of passengers who change to railway transportation. Corresponding measures can be taken on this basis, such as adjusting the train schedule around holidays. Further research can focus on how to adjust the transportation capacity of each connection mode.

The theory provides theoretical guidance for the evaluation of the departure efficiency of the off-station passenger flow of a comprehensive passenger transport hub, and is suitable for a comprehensive hub integrating multiple connection modes. It is noteworthy that the construction of our model is independent of the location and layout of various regions in the hub, which makes the model widely applicable. In contrast, as mentioned in section 2, although Tao et al. have fused the opinions of different experts, when the opinions of different experts are very different, the credibility of the results will be affected. In addition, Eshtaiwi’s model selects a large number of indicators to make up for the strong subjectivity, but the model is more suitable for the comparison of passenger flow between stations. Therefore, the hybrid model proposed in this paper has obvious advantages, which is convenient for managers to master the real-time passenger departure efficiency level of each connecting area, so as to find potential safety hazards and evacuate the large passenger flow in special periods in a timely manner and ensure the departure efficiency of the overall passenger flow of the hub. The research provides technical support and quantitative support for the management department to carry out real-time multimode capacity dispatching.

At present, the departure efficiency grade evaluated by the model has not been linked to the actual evacuation measures. Further research can focus on the large passenger flow under the background of different holidays, and predict the occurrence time of the large passenger flow phenomenon. At the same time, the corresponding capacity adjustment should be carried out according to the real-time departure efficiency of each transportation mode to put forward more constructive suggestions for the flow control of a hub. Furthermore, exploration can be taken on the departure impact during daily peak hours to make the research findings more applicable.

## Supporting information

S1 Table The index data of each connection area. (PDF)

## References

[1] Meng F, Yang L, Shi J, Jiang Z-Z, Gao Z. Collaborative passenger flow control for oversaturated metro lines: a stochastic optimization method. Transportmetrica A: Transport Science. 2021:1–40.

[2] [Terminology of multimodal passenger transportation hub]. China: Integrated transportation Standardization Technical Committee. CN-JT; 2016.

[3] Miller JD. A railroad terminal terminal evalution methodology (classification, yard) [dissertation]. Morgantown(WV): West Virginia University; 1985.

[4] Long JC, Vincent M, Mock DW. Bayport cruise terminal complex master plan, Port of Houston Authority, Houston, Texas. Ports 2007: 30 Years of Sharing Ideas: 1977–2007. p. 1–10.

[5] Andreatta G, Brunetta L, Righi L. Evaluating terminal management performances using SLAM: The case of Athens International Airport. Computers & Operations Research. 2007;34(6):1532–50.

[6] Deluka-Tibljas A, Karleusa B, Benac C. AHP methodology application in garage-parking facility location selection. Promet. 2011;23(4):303–13.

[7] Awasthi A, Chauhan SS, Goyal SK. A multi-criteria decision making approach for location planning for urban distribution centers under uncertainty. Mathematical and Computer Modelling. 2011;53(1–2):98–109.

[8] Bao LW, Mao CY, Qian J, Yang SD, Wang Q, Ieee, editors. Traffic connection simulation evaluation of high speed railway passenger hub based on VISSIM. IEEE 5th International Conference on Intelligent Transportation Engineering (ICITE); 2020 Sep 11–13; Beijing, PEOPLES R CHINA. NEW YORK: IEEE; 2020.

[9] Tang G, Zhao Z, Yu J, Sun Z, Li X. Simulation-based framework for evaluating the evacuation performance of the passenger terminal building in a Ro-Pax terminal. Automation in Construction. 2021;121:103445.

[10] Kallianiotis A, Papakonstantinou D, Arvelaki V, Benardos A. Evaluation of evacuation methods in underground metro stations. International Journal of Disaster Risk Reduction. 2018;31:526–34.

[11] Berrou JL, Beecham J, Quaglia P, Kagarlis MA, Gerodimos A. Calibration and validation of the Legion simulation model using empirical data. Pedestrian and evacuation dynamics 2005: Springer; 2007. p. 167–81.

[12] Chen JF, Liu C, Meng YY, Zhong MH. Multi-Dimensional evacuation risk evaluation in standard subway station. Safety Science. 2021;142:16.

[13] Jia HF, Cao XJ, Yang LL. Coordinated scheduling model for intermodal transit hubs based on GI/MK/1 queuing system. Journal of Central South University. 2015;22(8):3247–56.

[14] Lu J, Yang Z, Dong X, Zhu X. Design of timetable for airport coach based on ‘time–space’ network and passenger’s trip chain. Transport [Internet]. 2018 Jan.26;33(1):32–40.

[15] Yao LY, Xia XF, Sun LS. Transfer scheme evaluation model for a transportation hub based on vectorial angle cosine. Sustainability. 2014;6(7).

[16] Tao SY, Xie X. Evaluation of transfer system of high-speed railway station based on the integration of station city. proceedings of the 6th International Conference on Transportation Engineering; 2019: ICTE. p. 632–642.

[17] Lai XF, Teng J, Ling L. Evaluating public transportation service in a transit hub based on passengers energy cost. IEEE International Conference on Intelligent Transportation Systems (ITSC); 2020 Sep 20–23; ELECTR NETWORK: IEEE; 2020.

[18] Cheon SH, Lee C, Shin S. Data-driven stochastic transit assignment modeling using an automatic fare collection system. Transportation Research Part C-Emerging Technologies. 2019;98:239–254.

[19] Hora J, Dias TG, Camanho A, Sobral T. Estimation of origin-destination matrices under automatic fare collection: the case study of Porto transportation system. Transportation Research Procedia. 2017;27:664–671.

[20] Sipetas C, Keklikoglou A, Gonzales EJ. Estimation of left behind subway passengers through archived data and video image processing. Transportation Research Part C-Emerging Technologies. 2020;118: 102727. doi: 10.1016/j.trc.2020.102727 32834685 PMC7391996

[21] Hsu YW, Wang TY, Perng JW. Passenger flow counting in buses based on deep learning using surveillance video. Optik. 2020;202: 163675.

[22] Xuan RP, Xiong YX, Brietzke A, Marker S. Thermal infrared imaging based facial temperature in comparison to ear temperature during a real-driving scenario. J Therm Biol. 2021;96:102806. doi: 10.1016/j.jtherbio.2020.102806 33627258

[23] Guo Y, Agrawal S, Peeta S, Benedyk I. Safety and health perceptions of location-based augmented reality gaming app and their implications. Accident Analysis & Prevention. 2021;161:106354. doi: 10.1016/j.aap.2021.106354 34454283

[24] Vu L, Do Q, Nahrstedt K, editors. Jyotish: A novel framework for constructing predictive model of people movement from joint wifi/bluetooth trace. 2011 IEEE international conference on pervasive computing and communications (PerCom); 2011: IEEE; 2011. p.54–62. doi: 10.1109/PERCOMW.2011.5766968

[25] Versichele M, Neutens T, Delafontaine M, Van de Weghe N. The use of Bluetooth for analysing spatiotemporal dynamics of human movement at mass events: A case study of the Ghent Festivities. Applied Geography. 2012;32(2):208–220.

[26] Di Luzio A, Mei A, Stefa J, editors. Mind your probes: De-anonymization of large crowds through smartphone WiFi probe requests. IEEE INFOCOM 2016 The 35th Annual IEEE International Conference on Computer Communications; 2016: IEEE; 2016. p.1–9.

[27] Mehmood U, Moser I, Jayaraman PP, Banerjee A, Ieee, editors. Occupancy estimation using WiFi: a case study for counting passengers on busses. 5th IEEE World Forum on Internet of Things (IEEE WF-IoT); 2019 Apr 15–19; Univ Limerick, Elect & Comp Engn Dept, Limerick, IRELAND. NEW YORK: IEEE; 2019. p.165–170.

[28] Orsini F, Gastaldi M, Mantecchini L, Rossi R, Ieee, editors. Neural networks trained with WiFi traces to predict airport passenger behavior. 6th International Conference on Models and Technologies for Intelligent Transportation Systems (MT-ITS); 2019 Jun 05–07; Cracow Univ Technol, Krakow, POLAND. NEW YORK: IEEE; 2019. p.1–7.

[29] Zhou YY, Zhao MH, Sun LS, editors. Optimization of bottleneck facilities in subway stations based on WiFi data. 19th COTA International Conference of Transportation Professionals (CICTP)—Transportation in China 2025; 2019 Jul 06–08; Nanjing, PEOPLES R CHINA. NEW YORK: Amer Soc Civil Engineers; 2019. p.6287–6298.

[30] He YC, Xu J, Jia LM, Qin Y, Zhan KS, Zhang J, editors. Evaluation of emergency evacuation capacity of subway station based on M/G/c/c. 3rd International Conference on Electrical Engineering and Information Technologies for Rail Transportation (EITRT); 2017 Oct 20–22; Changsha, PEOPLES R CHINA. SINGAPORE: Springer-Verlag Singapore Pte Ltd; 2018.

[31] Tan ZJ, Xu M, Meng Q, Li ZC. Evacuating metro passengers via the urban bus system under uncertain disruption recovery time and heterogeneous risk-taking behaviour. Transportation Research Part C-Emerging Technologies. 2020;119: 102761.

[32] Qin Y, Zhang Z, Chen B, Xing Z, Liu J, Li J. Research on the prediction model for the security situation of metro station based on PSO/SVM. Journal of Intelligent Learning Systems and Applications. 2013; 5(4): 237–244.

[33] Guo Y, Li Y, Ch. Anastasopoulos P, Peeta S, Lu J. China’s millennial car travelers’ mode shift responses under congestion pricing and reward policies: A case study in Beijing. Travel Behaviour and Society. 2021;23:86–99.

[34] Alawad H, An M, Kaewunruen S. Utilizing an adaptive neuro-fuzzy inference system (ANFIS) for overcrowding level risk assessment in railway stations. Applied Sciences-Basel. 2020;10(15):5156.

[35] Sipetas C, Keklikoglou A, Gonzales EJ. Estimation of left behind subway passengers through archived data and video image processing. Transportation Research Part C-Emerging Technologies. 2020;118: 102727. doi: 10.1016/j.trc.2020.102727 32834685 PMC7391996

[36] Cornet S, Buisson C, Ramond F, Bouvarel P, Rodriguez J. Methods for quantitative assessment of passenger flow influence on train dwell time in dense traffic areas. Transportation Research Part C-Emerging Technologies. 2019;106:345–59.

[37] Daamen W, Lee Y-c, Wiggenraad P. Boarding and alighting experiments: overview of setup and performance and some preliminary results. Transportation Research Record. 2008;2042(1):71–81.

[38] Buchmüller S, Weidmann U, Nash A. Development of a dwell time calculation model for timetable planning. WIT Transactions on The Built Environment. 2008;103:525–534.

[39] Tesoriere G, Campisi T, Canale A, Severino A, Arena F, editors. Modelling and simulation of passenger flow distribution at terminal of catania airport. International Conference of Computational Methods in Sciences and Engineering (ICCMSE); 2018 Mar 14–18; Thessaloniki, GREECE. MELVILLE: Amer Inst Physics; 2018.

[40] Zhu YW, Koutsopoulos HN, Wilson NHM. Passenger itinerary inference model for congested urban rail networks. Transportation Research Part C-Emerging Technologies. 2021;123: 102896.

[41] Shirakawa M, editor The analysis and model design of aircraft vertical profile using factor analysis. Proceedings of 13th International Conference on Digital Signal Processing; 1997: IEEE. p.699–702 vol.2.

[42] Souza W, Aoki A, Neto A, editors. Factor analysis in data mining applied for recognition and classification pattern for smart grid. 2013 IEEE PES Conference on Innovative Smart Grid Technologies (ISGT Latin America); 2013: IEEE. p.1–8.

[43] Du S, Xiong L, Ding W. Comprehensive evaluation of urban rail transit network planning based on green transportation principle. Journal of Southwest Jiaotong University. 2006;41(3):284–9.

[44] Wu Y, Kang J, Mu JY. Assessment and simulation of evacuation in large railway stations. Building Simulation. 2021;14(5):1553–1566.

[45] Eshtaiwi M, Badi I, Abdulshahed A, Erkan TE. Determination of key performance indicators for measuring airport success: A case study in Libya. Journal of Air Transport Management. 2018;68:28–34.

[46] Braglia M, Castellano D, Gabbrielli R. A novel game theory based exit selection model in emergency conditions. Advances in Complex Systems. 2013;16(7):1350018.

[47] Zhang GW, Huang D, Zhu GQ, Yuan GL. Probabilistic model for safe evacuation under the effect of uncertain factors in fire. Safety Science. 2017;93:222–229.

[48] Zhang ZY, Zhao XJ, Qin Y, Si HY, Zhou LX. Interval type-2 fuzzy TOPSIS approach with utility theory for subway station operational risk evaluation. Journal of Ambient Intelligence and Humanized Computing. 2021:1–15.

[49] Kepaptsoglou K, Karlaftis MG, Gkountis J. A fuzzy AHP model for assessing the condition of metro stations. Ksce Journal of Civil Engineering. 2013;17(5):1109–1116.

[50] Elzarka HM, Yan HY, Chakraborty D. A vague set fuzzy multi-attribute group decision-making model for selecting onsite renewable energy technologies for institutional owners of constructed facilities. Sustainable Cities and Society. 2017;35:430–439.

[51] Geng XL, Chu XN, Zhang ZF. A new integrated design concept evaluation approach based on vague sets. Expert Systems with Applications. 2010;37(9):6629–6638.

[52] Subedi S, Pauls E, Zhang YD. Accurate localization and tracking of a passive RFID reader based on RSSI measurements. IEEE Journal of Radio Frequency Identification. 2017;1(2):144–54.

[53] Yu W, Ye XF, Chen J, Yan XC, Wang T. Evaluation indexes and correlation analysis of origination-destination travel time of Nanjing metro based on complex network method. Sustainability. 2020;12(3):21.

[54] Lin SF, Wei QW, Xia SB, Iop, editors. Research on the evaluation index system of intelligent railway passenger station. International Symposium on Power Electronics and Control Engineering (ISPECE); 2018 Dec 28–30; Xian Univ Technol, Xian, PEOPLES R CHINA. BRISTOL: Iop Publishing Ltd. 2019. 1187(5):052.

[55] Bai Y, Hu QY, Ho TK, Guo HY, Mao BH. Timetable optimization for metro lines connecting to intercity railway stations to minimize passenger waiting time. IEEE Transactions on Intelligent Transportation Systems. 2021;22(1):79–90.

[56] Sun LJ, Jin JG, Lee DH, Axhausen KW, Erath A. Demand-driven timetable design for metro services. Transportation Research Part C-Emerging Technologies. 2014;46:284–299.

[57] Cox T, Houdmont J, Griffiths A. Rail passenger crowding, stress, health and safety in Britain. Transportation Research Part A: Policy and Practice. 2006;40(3):244–58.

