# Supplementary material for: Departure efficiency evaluation of a comprehensive transport hub based on Wi-Fi probe data and a multilayer hybrid model
Source: PLoS One. 2022 Mar 4;17(3):e0264473. doi: 10.1371/journal.pone.0264473 (PMC8896716; doi:10.1371/journal.pone.0264473)
Supplement: S1 Table — (PDF) [file pone.0264473.s001.pdf]

S1 Table. the Index Data of Each Connection Area

Date	Time	Hub Name	Stranded Passengers				Wait Time/min				Departure Time/min			
			rail	bus	taxi	couch	rail	bus	taxi	coach	rail	bus	taxi	coach
2019/9/30	8:00	Chongqing north railway station	69	8	3	24	5.3	8.9	13.1	40.7	10.7	19.5	15.5	65.4
2019/9/30	8:30	Chongqing north railway station	146	68	9	26	5.3	8.2	11.0	41.2	8.3	17.0	9.4	66.1
2019/9/30	9:00	Chongqing north railway station	134	77	4	44	5.4	8.2	9.5	40.3	9.4	14.5	7.4	78.7
2019/9/30	9:30	Chongqing north railway station	198	86	11	59	5.6	8.6	9.0	39.5	10.6	14.8	7.6	43.1
2019/9/30	10:00	Chongqing north railway station	167	68	8	51	5.7	8.5	9.1	35.0	11.1	14.7	8.5	43.0
2019/9/30	10:30	Chongqing north railway station	162	110	13	77	6.0	8.1	8.2	33.4	11.9	17.6	10.3	58.9
2019/9/30	11:00	Chongqing north railway station	130	39	5	88	6.3	8.3	8.6	39.0	12.7	18.9	14.7	81.7
2019/9/30	11:30	Chongqing north railway station	150	78	15	106	6.5	8.3	8.0	37.9	13.1	15.9	15.2	77.6
2019/9/30	12:00	Chongqing north railway station	209	113	56	130	6.7	8.1	7.2	33.7	13.6	15.9	12.8	67.6
2019/9/30	12:30	Chongqing north railway station	143	66	10	101	6.8	8.0	7.1	34.0	14.6	19.2	14.7	68.0
2019/9/30	13:00	Chongqing north railway station	192	134	4	152	6.6	7.9	7.4	32.8	13.6	15.9	15.1	58.0
2019/9/30	13:30	Chongqing north railway station	207	107	11	136	6.6	8.1	7.1	32.4	13.4	14.3	12.8	63.5
2019/9/30	14:00	Chongqing north railway station	205	125	16	108	6.5	8.0	6.7	32.3	14.6	14.3	13.4	68.5
2019/9/30	14:30	Chongqing north railway station	283	209	63	106	6.4	8.0	6.7	31.4	14.9	16.5	15.0	57.9
2019/9/30	15:00	Chongqing north railway station	208	93	33	176	6.5	8.2	7.2	29.9	15.6	18.6	17.7	51.1
2019/9/30	15:30	Chongqing north railway station	268	167	63	180	6.4	8.0	7.5	31.1	15.1	16.7	16.0	47.3
2019/9/30	16:00	Chongqing north railway station	256	120	50	227	6.6	7.9	8.0	31.8	15.4	16.6	21.3	50.7
2019/9/30	16:30	Chongqing north railway station	293	147	52	222	6.7	7.9	8.3	32.6	16.5	16.1	22.7	63.5
2019/9/30	17:00	Chongqing north railway station	219	74	11	196	6.6	8.0	8.4	33.9	16.4	16.7	21.7	68.9
2019/9/30	17:30	Chongqing north railway station	192	84	4	145	6.6	8.0	8.6	34.2	15.9	17.5	19.3	70.4
2019/9/30	18:00	Chongqing north railway station	223	93	15	134	6.6	8.1	8.6	35.1	12.1	18.2	12.0	73.7
2019/9/30	18:30	Chongqing north railway station	296	242	49	103	6.6	8.2	8.4	36.2	13.6	16.5	13.1	70.9
2019/9/30	19:00	Chongqing north railway station	264	191	72	117	6.5	8.4	8.6	37.4	16.4	18.1	18.3	64.8
2019/9/30	19:30	Chongqing north railway station	283	207	88	134	6.6	8.4	8.6	38.1	18.2	17.5	19.4	56.9
2019/9/30	20:00	Chongqing north railway station	301	107	34	101	6.7	8.5	8.7	38.5	19.3	20.1	20.9	55.5
2019/9/30	20:30	Chongqing north railway station	253	167	90	73	6.7	8.5	8.7	37.9	18.4	21.0	18.7	46.9
2019/9/30	21:00	Chongqing north railway station	248	146	60	42	6.7	8.5	9.0	38.2	16.9	16.7	18.7	58.5
2019/9/30	21:30	Chongqing north railway station	213	146	73	11	6.8	8.4	9.2	39.1	15.6	15.9	23.9	57.2
